# Joint torque variability and repeatability during cyclic flexion-extension of the elbow

**DOI:** 10.1186/s13102-016-0033-1

**Published:** 2016-04-11

**Authors:** Laurent Ballaz, Maxime Raison, Christine Detrembleur, Guillaume Gaudet, Martin Lemay

**Affiliations:** 1 Department of kinanthropology, Université du Québec à Montréal, Montreal, Qc Canada; 2 Department of mechanical engineering, École Polytechnique de Montréal, Montreal, Qc Canada; 3 Research & Engineering Chair Applied to Pediatrics (RECAP), Marie Enfant Rehabilitation Centre (CRME) – Research Center – Sainte-Justine UHC, and École Polytechnique de Montréal, Montreal, Qc Canada; 4 Institute of NeuroSciences (IoNS), Université catholique de Louvain, Bruxelles, Belgium; 5 CRME – Research Center, Office GR-123, 5200, East Bélanger Street, H1T 1C9 Montréal, QC Canada

**Keywords:** Modeling, Inverse dynamics, Kinematic solidification, Elbow joint torques, Variability, Repeatability

## Abstract

**Background:**

Joint torques are generally of primary importance for clinicians to analyze the effect of a surgery and to obtain an indicator of functional capability to perform a motion. Given the current need to standardize the functional evaluation of the upper limb, the aim of this paper is to assess (1) the variability of the calculated maximal elbow joint torque during cyclic elbow flexion-extension movements and (2) participant test-retest repeatability in healthy young adults. Calculations were based on an existing non-invasive method including kinematic identification and inverse dynamics processes.

**Methods:**

Twelve healthy young adults (male *n* = 6) performed 10 elbow flexion-extension movement carrying five different dumbbells (0, 1, 2, 3 and 4 kg) with several flexion-extension frequencies (½, ^1^/_3_, ¼ Hz) to evaluate peak elbow joint torques.

**Results:**

Whatever the condition, the variability coefficient of trial peak torques remained under 4 %. Bland and Altman plot also showed good test-retest, whatever the frequency conditions for the 0, 1, 2, and 3 kg conditions.

**Conclusion:**

The good repeatability of the flexion-extension peak torques represents a key step to standardize the functional evaluation of the upper limb.

## Background

In many musculoskeletal diseases muscular weakness leads to functional disability and decreased quality of life. For therapists, it is important to assess and quantify muscle strength in order to choose the most appropriate treatment or to evaluate therapy effects [1, 2]. Joint torques are generally of primary importance for clinicians to analyze the effect of a surgery on symmetry and comfort, and to obtain an indicator of functional capability to perform a motion. Joint torques are very often analyzed in patients with osteoarthritis (e.g.: [3, 4]) or scoliosis (e.g.: [5, 6]). Especially at the elbow, the change in elbow torque is an indicator of incremental release of the brachioradialis insertion footprint, for surgeons performing open reduction or internal fixation of distal radius fractures [7]. For physio/ergo-therapists, the elbow torque is an indicator of functional capability to perform a motion, e.g. in stroke patients, and a control variable for assistive devices developed for these patients [8]. In the rehabilitation field, strength is assessed though the measurement of the maximal joint torque [9–11], which represents the resultant action of all muscles crossing the joint, but do not provide each muscle force contribution. Studies have shown the potential of musculoskeletal simulation tools to determine the contribution of each muscle crossing a joint during movement which was otherwise impractical or impossible to obtain experimentally [12]. According to the clinical relevance and accuracy of the used method, such quantification would help clinicians to target the best therapeutic solution. Indeed, computational model could give the opportunity to predict the effect of the muscle property modifications on joint torque production [13]. For example, the effect of antagonist muscle release (e.g.: spasticity treatment) on joint torque production could be anticipated.

The upper limb function is of utmost importance in improving the quality of life and enhancing functional independence. Especially, elbow flexion movement has been related to motor impairment and performance [14]. Thus, accurate modeling of elbow muscle involvement could provide an interesting tool to better understand the movement limitation. Within this process of calculating the muscle forces, joint torque is an essential intermediate variable [15–17]. Moreover, precise and repeatable quantification of the upper limb joint torque is of major importance for numerous applications (e.g. [18–20]) including exoskeletons and interactive rehabilitation devices development (e.g. [18, 21]), the understanding of the mechanisms resulting in joint rigidity (e.g. [22, 23]), or the impact of joint co-contraction on joint constraint (e.g. [17, 24]).

However, it is not always obvious to obtain accurate joint torque results that could be usefully exploited in model [25–27]. Applied to human motion analysis, several parameters can be a source of error. The major problems are linked to the inverse dynamic solution repeatability, which is affected by both the data processing and the experimental procedure. More specifically, in a top down approach, inaccuracy in movement coordinate data, joint centre of rotation location, and kinematic data processing can impact on inverse dynamics solution [25]. Indeed, using marker-based optical motion capture systems, marker misallocation and skin movement greatly influence joint centre localisation [28, 29]. The inertia parameters of the body segments can also influence inverse dynamic solution [30]. Lastly, the estimate of internal efforts, i.e. joint torques and muscle forces, is particularly sensitive to accelerations [31–33]. As a result, kinematic data analysis is also of greatest importance and mainly impact inverse dynamic results. Riemer et al. found that these various inaccuracies can result in uncertainties of estimated joint torques ranging from 6 % to 232 % of the peak torque during gait. As suggested in the literature however, more accurate results can be obtained with corrected kinematics based on a kinematic identification process, named solidification procedure [34], compared to inverse dynamics using either raw kinematic data, smoothing or low-pass filtering [35–37].

Additionally, in order to use inverse dynamics to follow patient progress, the experimental procedure should (1) allow the spontaneous adaptation of the participant to perform the task (e.g.: minimally constraint movement) and (2) result in within-subject test-retest task repeatability, according to the kinematic and dynamic movement parameters used in the model.

In light of this information, we have developed a model which quantifies the contribution of muscles crossing the elbow joint during flexion and extension movements [17] in order to use it as a clinical tool. The model-based process includes two consecutive steps: a kinematic identification based on procedure of solidification [34], combined with inverse kinematics and an inverse dynamics process that provides the elbow joint net torque (for more details, see [17]). As a first step to test the accuracy of the model, the aim of the present study is (1) to assess the maximal elbow joint torque variability during cyclic elbow flexion extension movements and (2) to assess participant test-retest repeatability in healthy young adults.

## Methods

### Participants

Twelve healthy young adults (age = 23 ± 2; male *n* = 6) were included in the present study. Exclusion criteria were known musculoskeletal or orthopaedic pathology, on the basis of a questionnaire in participants. The study was approved by the Research Ethics Board of Ste-Justine Hospital, Montreal, Canada (Ethics case #3362). A written informed consent was obtained from participants. The research was in compliance with the Helsinki Declaration.

### Procedure

#### Experimental set-up

**Fig 1:**
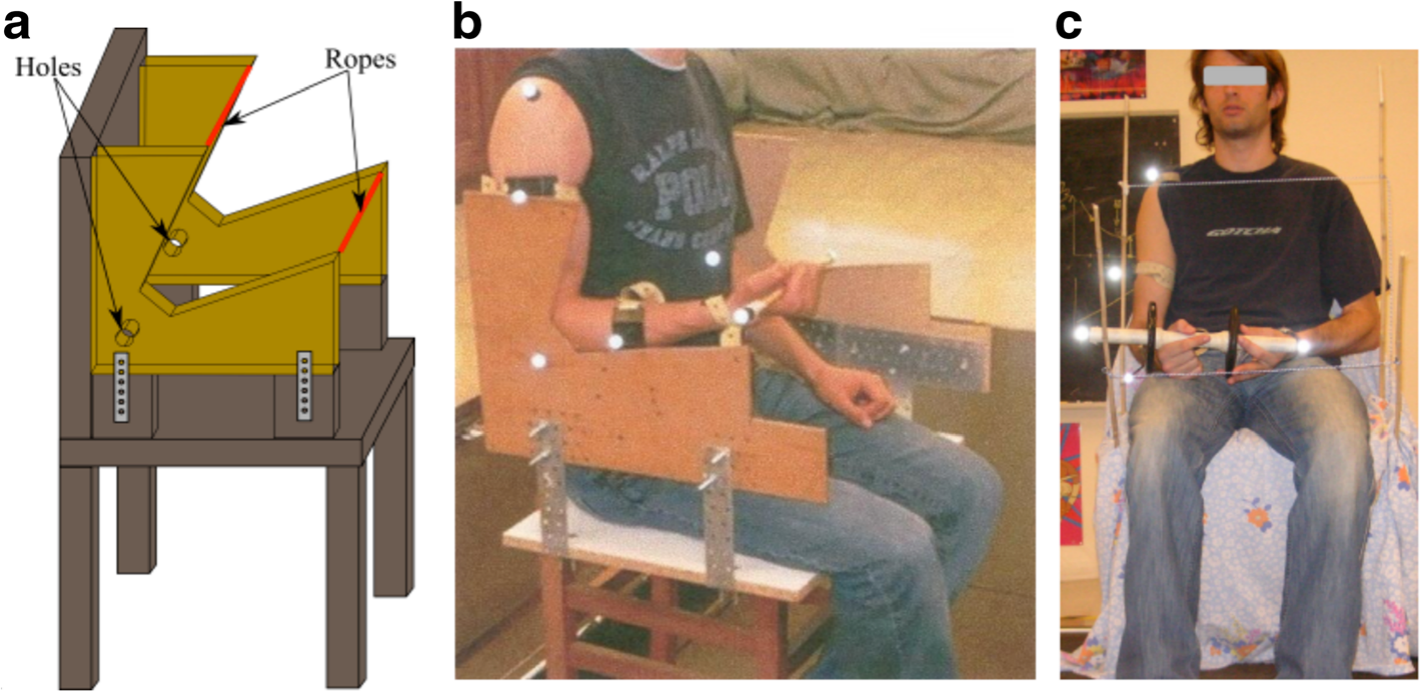
Experimental chair designed to perform elbow flexion/extension in the sagittal plane. Legend: **a** Design plan of the chair, featuring the ropes that limit the motion amplitude and the holes into which the elbow optokinetic sensor is inserted to avoid the elbow motion; **b** Side view of the chair, pointing out the elbow optokinetic sensor is inserted to avoid the elbow motion; **c** Front view of the chair, pointing out the ropes that limit the motion amplitude

The experiments were conducted on cyclic elbow flexion-extension movement with the upper arm maintained vertical. As illustrated in Fig. 1, an experimental chair was designed to enable standardized motion of elbow flexion-extension in the sagittal plane. The person depicted in Fig. 1c gave a special consent to publish this one. Particularly, our incentive was to minimize the elbow joint motion during the task, but without mechanically blocking it, to highlight the behaviour of only one joint, i.e. the elbow. Consequently, right elbow optokinetic sensors were inserted in specific holes created on the side of the chair rest (Fig. 1a). Further, to limit the range of the flexion-extension motion (approximately 50°), 'sensitive' stops were placed to keep the movement between 70 and 120 degrees of flexion (Fig. 1a). This arc (70–120) was chosen because it corresponds to range of movement involve in many functional tasks [38]. The chair was adapted in height and depth in order to seat the participant with their hips and knees flexed at 90 degrees, and the right arm placed vertically downward. The participants were equipped with optokinetic sensors, placed on the following anatomical landmarks: the acromion, the middle of the arm (technical marker), the lateral epicondyle, the middle of the forearm (technical marker), the radial styloid, and both extremities of the dumbbells. This placement was set to enable the three-dimensional kinematic reconstruction of the upper limb and the dumbbell. The displacement of the markers was filmed by six infrared cameras (Elite-BTS, Milano, Italy) cadenced at 100 Hz.

### Participant instructions

During experimentation, the participant sat on the chair. The participants were asked to perform 10 cycles of flexion-extension, following the rhythm of a given metronome, with and without dumbbells. Participants had to keep the shoulder and elbow joint as motionless as possible and the dumbbell axis horizontal. Participants were involved a few minutes with the dumbbells, before beginning the experiments.

The participants had to perform ten elbow flexion-extension movements with five different masses: 0, 1, 2, 3 and 4 kg, and at three motion frequencies, 0.5 Hz (i.e*.* a cycle in 2 seconds), 0.33 Hz (1 cycle in 3 seconds) and 0.25 Hz (1 cycle in 4 seconds). The order of the masses and frequencies was drawn randomly by the operator. Each male participant performed the whole experimental protocol twice in order to assess test and retest reproducibility of the joint torques. The retests were performed approximately 20 min after the tests, without removing the kinematic sensor.

### Joint torque quantification process

Using the measurements of kinematic sensors, a 3D multibody model of the human body [17] provides the elbow joint torques via these three consecutive steps:

1. The full model joint kinematics: the system is modeled as a constrained multibody system, using kinematic loops.
2. The joint kinematic identification: the joint coordinates *q*, velocities q̇ and accelerations q̈ are numerically determined by an optimization process that estimates the joint coordinates of the multibody model that best fit the experimental joint positions.
3. The inverse dynamics: using recursive Newton-Euler formalism, a 3D multibody model [17] provides the vector *Q*_*inv*_ of joint forces and torques during movement as follows:

1 $$ {Q}_{inv}=f\left(q,\ \overset{.}{\mathrm{q}},\ \ddot{\mathrm{q}},{F}_{ext},{M}_{ext},g\right) $$

where *f* is a function of the kinematics *q*, q̇, q̈ and represents the inverse dynamical model of the human body, on the basis of the external forces *F*_*ext*_ and torques *M*_*ext*_ applied to the system, and also gravity *g*. The inertia parameters of the body segments have been defined using the Table from de Leva [39].

These equations were symbolically generated by the ROBOTRAN software [40], UCL, which allows us to straightforwardly interface these equations with any numerical process, such as the optimization process presented above and the time simulation of the trials.

### Statistical analysis

Data was reported as mean (standard deviation) (SD). Normality of the distributions was determined using the Kolmogorov–Smirnov test. For each frequency (0.5, 0.3, and 0.25 Hz) and mass (0, 1, 2, 3, 4 kg), the peak torque variability within each trial was assessed by computing the coefficient of variation (%CV). The aim of this intra-test variability analysis was to enable to average the peak torques of each trial for the repeatability analysis. Paired t-tests were performed to detect possible systematic bias between test and retest trial. The possibility of heteroscedasticity was examined on the basis of the Pearson product-moment correlation (r) between the mean and the absolute differences. If the correlation coefficient was significant the data were considered as heteroscedastic [41]. Bland and Altman plots and limits of agreement analyses were also calculated to determine whether peak torque is in agreement between tests and retest trial [42]. This method (Bland & Altman, 1986) was extensively used in different research fields in test-retest studies [43–46] and is suitable in the case of the present study [41]. A corrected standard deviation of differences for repeated measurements, *SD*_*corrected*_ = √(2●*SD*^2^), was used based on Bland and Altman (1986) [42]. Statistical analysis was performed using SPSS 17.0 (IBM, Chicago, USA).

## Results

In each condition, the peak torque values were normally distributed (Kolmogorov–Smirnov test, *p* > 0.05).

### Intra-test variability

Whatever the test conditions, the variation coefficient of the peak torque ranged between 0.8 and 4 % (see Table 1).

**Table 1:** Peak torque coefficients of variation within trial

	Peak torque CV (*n* = 12)	
Test conditions mass (Kg)/Frequency (Hz)	Mean (SD)	95 % confidence interval
0/0.25	1.3 (1.1)	0.6-2.0
0/0.33	0.8 (0.4)	0.5-1.0
0/0.5	0.9 (0.5)	0.6-1.2
1/0.25	3.9 (3.9)	1.4-6.4
1/0.33	4.0 (3.1)	2.1-6.1
1/0.5	3.0 (2.9)	1.2-4.9
2/0.25	1.2 (1.1)	0.5-2.0
2/0.33	1.3 (1.5)	0.3-2.3
2/0.5	1.1 (0.6)	0.7-1.5
3/0.25	0.8 (0.4)	0.6-1.1
3/0.33	1.0 (1.0)	0.4-1.7
3/0.5	1.3 (0.9)	0.7-1.9
4/0.25	1.3 (0.5)	1.0-1.8
4/0.33	1.1 (0.9)	0.5-1.7
4/0.5	0.9 (0.3)	0.7-1.2
Mean	1.6	
SD	1.1	

Legend: *CV* coefficient of variation, *SD* standard deviation

### Test-retest repeatability

**Fig 2:**
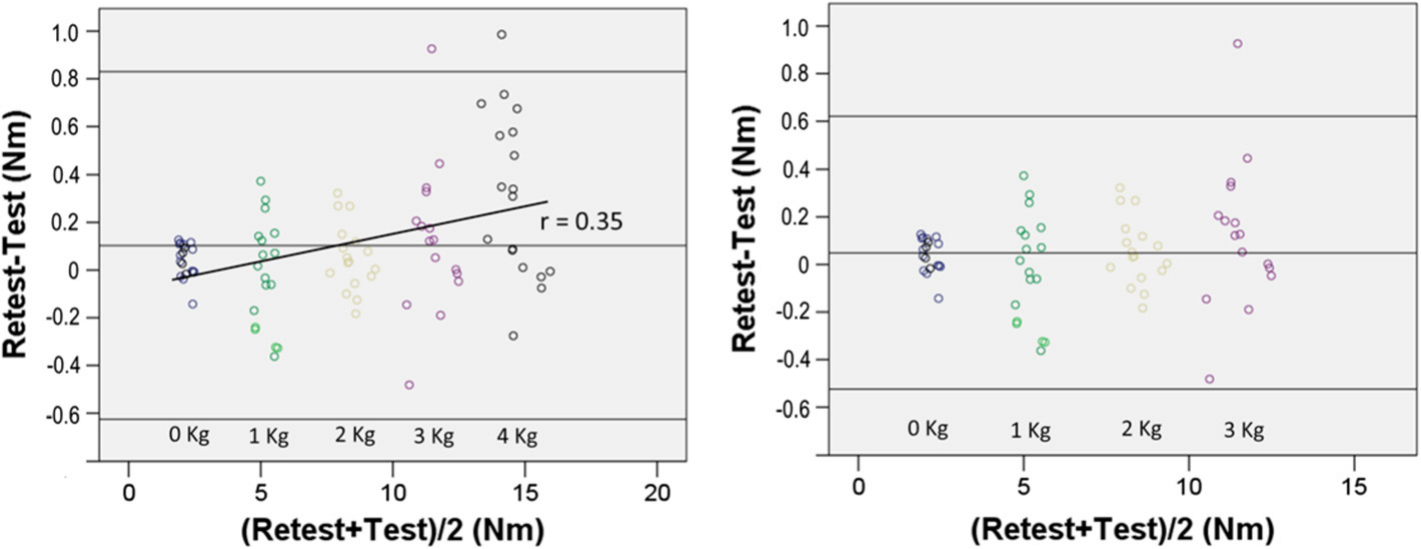
Bland and Altman plot for peak torque repeatability. Legend: Bland and Altman plot of the difference between test and retest peak torque values. The left panel illustrates that the homoscedasticity assumption would be violated if the 4 kg condition were included in the analysis (a correlation exists, *p* < 0.05). The right panel illustrates that the homoscedasticity assumption is met (no correlation exists, *p* > 0.05) if the 4 kg condition is dropped

Test-retest repeatability was performed with the male participants (*n* = 6). Whatever the condition, test and retest values were not significantly different (*p* > 0.05). Considering that the assumption of homoscedasticity was not met when the 4 kg conditions were included in the analysis, the conditions involving 4 kg were no longer considered in the present study. Whatever the other test conditions, the limits of agreement were -0.52 Nm to 0.62 Nm, which represent a variation of 8.5 % of the averaged peak torques (6.7 Nm) around the mean test-retest difference (See Bland and Altman plots, Fig. 2, right panel).

**Fig 3:**
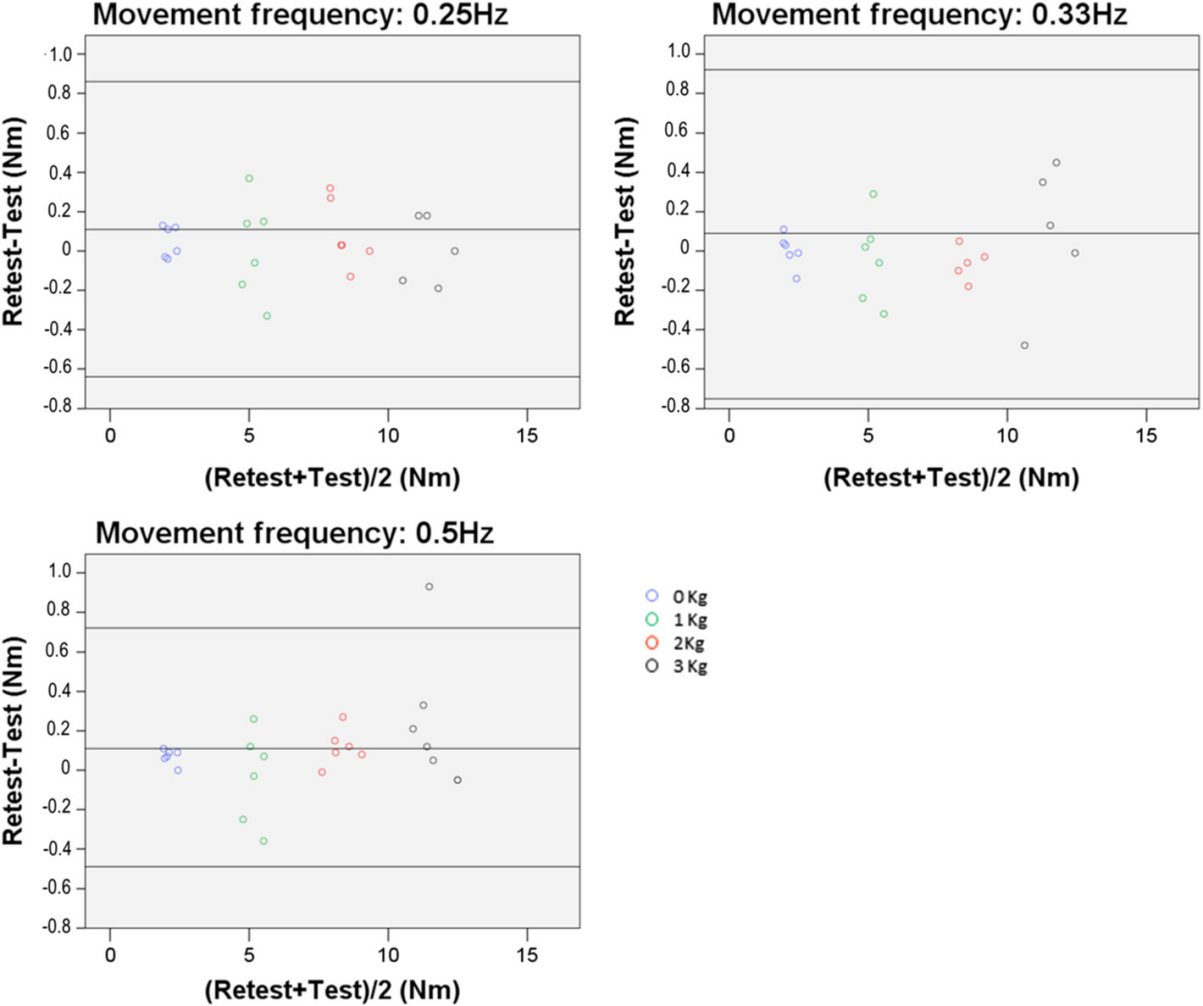
Bland and Altman plots for peak torque repeatability at each frequency condition. Legend: Bland and Altman plots of the difference between test and retest peak torque values for each frequency condition

Whatever the mass condition, with a frequency of 0.25, 0.33, and 0.5 Hz, the limits of agreement values were -0.64 Nm to 0.86 Nm, -0.75 Nm to 0.92 Nm, and -0.49 Nm to 0.72 Nm, which represent a variation of 9.1, 9.9, and 7.2 % of the averaged peak torques (8.3 Nm, 8.4 Nm, and 8.3 Nm) around the mean test-retest differences, respectively (see Fig. 3).

**Fig 4:**
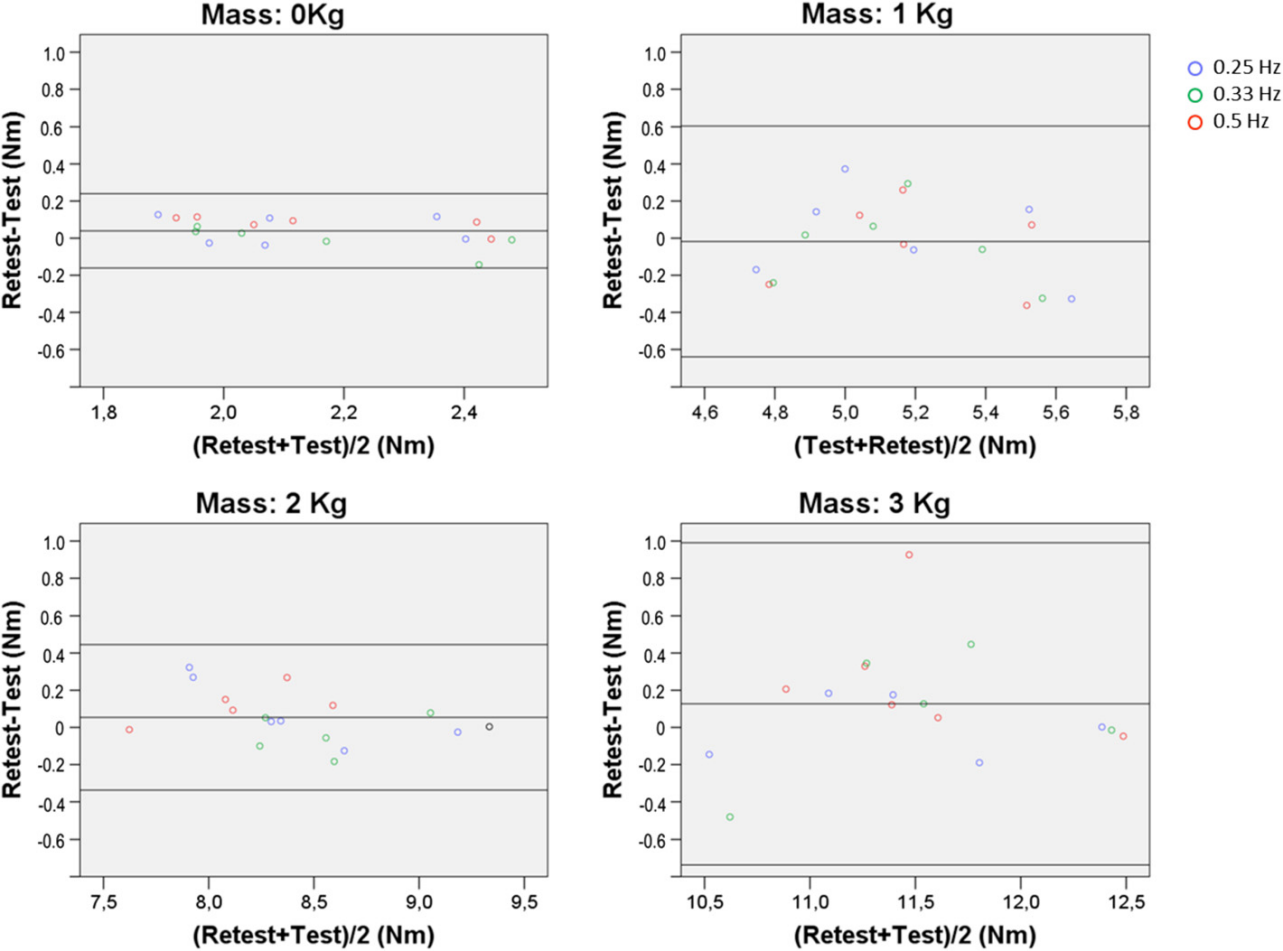
Bland and Altman plots for peak torque repeatability at each mass condition. Legend: Bland and Altman plots of the difference between test and retest peak torque values for each mass condition

Whatever the frequency condition, with a mass of 0, 1, 2, and 3 kg, the limits of agreement values were -0.16 Nm to 0.24 Nm, -0.64 Nm to 0.60 Nm, -0.34 Nm to 0.44 Nm, -0.74 Nm to 0.99 Nm, which represent a variation of 9.3, 12, 4.6, and 7.6 % of the averaged peak torques torques (2.2 Nm, 5.2 Nm, 8.5 Nm, and 11.4 Nm) around the mean test-retest differences, respectively (see Fig. 4).

## Discussion

This study showed that the data processing and the experimental procedure implemented in the present study resulted in a low within-trial variability, i.e. a low variability inside each trial, and a good within-participant test-retest repeatability, i.e. a good repeatability between tests of the same participant, of the elbow peak torque in typically developing young adults. As shown by the limit of agreements, expressed as a percentage of the averaged peak torque, the result repeatability was equivalent whatever the frequency, amongst 0.25, 0.33, and 0.5 Hz, or the load, amongst 0 1, 2, and 3 kg, imposed during the movement.

This study highlighted that the 4 kg resulted in a more important variability compared to the lower masses. Based on this observation, it can be assumed that increasing the mass higher than 4 kg would result in a more important variability that would not be appropriated to evaluate the joint torques. On the contrary, using lower masses, such as 0 kg, are recommended for the good repeatability, and certainly do not imply fatigue, especially in female participants.

In summary, to evaluate muscle efforts in the rehabilitation field, the repeatability of the model at low frequencies and with light loads was a key result. In patients with neurological disorder, muscular strength and movement velocity is potentially very low depending on their functional capacity. As supported by the Bland and Altman analysis (Fig. 3), at low frequency (0.25Hz) the limit of agreement represented 9.1 % of the averaged peak torque, and considering the condition without dumbbells, the limit of agreement represented 9.3 % of the averaged peak torque. Even if the literature still has no consensus on the clinically important difference in elbow torque for humans, because this torque relates to each joint and each motion, Laitenberger et al. (2015) [47] reported an elbow torque variability up to 24 % in healthy subjects, which confirms that the obtained repeatability of 8.5 % when all test conditions are viewed together (Fig. 2) is relevant compared to the magnitude of this measurement. As described earlier, kinematic data processing, marker misallocation and skin movement could greatly influence joint centre localisation [28, 29] and in turn greatly impact inverse dynamic solution repeatability [25]. Riemer et al. found that these various inaccuracies can result in uncertainties of estimated joint torques ranging from 6 % to 232 % of the peak torque during gait. The methodology used in the present study in terms of kinematic data processing, based on solidification procedure [34], was adequate to result in a good within-participant test-retest repeatability. At the same time, these results showed that the device used (Fig. 4) was adequate to obtain a repeatable elbow flexion-extension maximal torque.

Several limits were inherent with this study. First the repeatability of the data processing and the experimental procedure was tested with a limited number of participants. Nevertheless, many conditions were tested (frequency*mass), resulting in a test-retest repeatability analysis based on 90 trials. The test-retest repeatability analysis was performed only in male because fatigue could be more present in female compared to male participants. Secondly, the present study included healthy participants, the repeatability of the data processing and the experimental procedure implemented in the present study should be tested for each targeted disease. Thirdly, the repeatability of the model has been tested without removing the maker. A Further study is required to test the reproducibility with markers replacement because markers location could impact the inverse dynamic solution. Fourthly, the gender effect on repeatability was not studied and could be a further perspective. Fifthly, the trials were randomised and a 3 min. rest period was allocated between the trials, as recommended by Kollmitzer et al. (1999) [48] to avoid the muscle fatigue effect in the context of a sub-maximal effort of the upper limb. However, it is never excluded that either a fatigue or a time effect may have influenced the CV results. Especially concerning the higher CV reported for the 1 kg condition, we believe that this results from the method variation that might also be seen in other conditions. Nevertheless, the CV reported for the 1 kg condition remains low, even if it represents twice the CV reported in the other conditions. Sixthly, a method with a good reproducibility does not necessarily guarantee an accurate estimation of joint torques. Reproducibility is a necessary feature that is complementary to the accuracy, guaranteeing that the results will be similar for any trial. Let us remind that it is still not possible today to check the accuracy of the joint torques in a non-invasive way, even if this information is of primary importance for clinicians to analyze the effect of a surgery and to obtain an indicator of functional capability. Being aware of these limitations, the incentive of this paper was to analyse the quality of our joint torque quantification. The present inverse dynamical model of the human body, necessarily preceded by a kinematic identification of the model configurations, is proposed as a satisfying method to estimate the joint efforts in dynamical context. This problem being deterministic, *Q*_*inv*_ becomes a sufficiently accurate result that can be exploited as a reference for the optimization process that attempts to solve the muscle force redundancy. These results represent the first step leading to the development of an accurate assessment of elbow muscle strength in clinical environment. The ability of giving accurate elbow joint net torques during motion, without requiring an important computational cost, is the main benefit of this method. Based on these results, multibody model refinement and clinical analysis will be implemented in further studies.

## Conclusion

The aim of this study was to assess the peak torque elbow variability and repeatability. Whatever the flexion-extension movement conditions imposed, within-trial peak torque variability was low and within-participant test-retest repeatability of the elbow joint torques resulted in good agreement. This method is promising for potential clinical applications and can be used as a basis for further comparison between efforts quantification methods or refined multibody models in the human body during motion.

### Availability of data and materials

The authors’ Research Ethics Board did not allow to publicly share the data and materials of this study. However, these data and materials will be available upon request to the corresponding author and in accordance with the Research Ethics Board.
